# Analysis of Non-Volatile Chemical Constituents of Menthae Haplocalycis Herba by Ultra-High Performance Liquid Chromatography-High Resolution Mass Spectrometry

**DOI:** 10.3390/molecules22101756

**Published:** 2017-10-19

**Authors:** Lu-Lu Xu, Jing-Jing Xu, Kun-Rui Zhong, Zhan-Peng Shang, Fei Wang, Ru-Feng Wang, Le Zhang, Jia-Yu Zhang, Bin Liu

**Affiliations:** 1 School of Chinese Pharmacy, Beijing University of Chinese Medicine, Beijing 100029, China; xll@bucm.edu.cn (L.-L.X.); chichuxieyang@163.com (J.-J.X.); markselby1992@163.com (K.-R.Z.); zpshang1206@163.com (Z.-P.S.); feiwang1016@126.com (F.W.); wangrufeng@tsinghua.org.cn (R.-F.W.); gail3123@sina.com (L.Z.); 2 Beijing Research Institution of Chinese Medicine, Beijing University of Chinese Medicine, Beijing 100029, China

**Keywords:** Menthae Haplocalycis herba, UHPLC-ESI-MS/MS, non-volatile constituents, target isolation

## Abstract

Menthae Haplocalycis herba, one kind of Chinese edible herbs, has been widely utilized for the clinical use in China for thousands of years. Over the last decades, studies on chemical constituents of Menthae Haplocalycis herba have been widely performed. However, less attention has been paid to non-volatile components which are also responsible for its medical efficacy than the volatile constituents. Therefore, a rapid and sensitive method was developed for the comprehensive identification of the non-volatile constituents in Menthae Haplocalycis herba using ultra-high performance liquid chromatography coupled with linear ion trap-Orbitrap mass spectrometry (UHPLC-LTQ-Orbitrap). Separation was performed with Acquity UPLC^®^ BEH C_18_ column (2.1 mm × 100 mm, 1.7 μm) with 0.2% formic acid aqueous solution and acetonitrile as the mobile phase under gradient conditions. Based on the accurate mass measurement (<5 ppm), MS/MS fragmentation patterns and different chromatographic behaviors, a total of 64 compounds were unambiguously or tentatively characterized, including 30 flavonoids, 20 phenolic acids, 12 terpenoids and two phenylpropanoids. Finally, target isolation of three compounds named Acacetin, Rosmarinic acid and Clemastanin A (first isolated from Menthae Haplocalycis herba) were performed based on the obtained results, which further confirmed the deduction of fragmentation patterns and identified the compounds profile in Menthae Haplocalycis herba. Our research firstly systematically elucidated the non-volatile components of Menthae Haplocalycis herba, which laid the foundation for further pharmacological and metabolic studies. Meanwhile, our established method was useful and efficient to screen and identify targeted constituents from traditional Chinese medicine extracts.

## 1. Introduction

As there is growing interest in the use of traditional Chinese medicines (TCMs), systematic screening and identification of chemical components is essential for revealing the material basis of their therapeutic effects and ensuring their safety [1]. However, TCM extracts composed of multi-components are difficult to be comprehensively analyzed. Ultra-high performance liquid chromatography coupled with electrospray ionization tandem mass spectrometry (UHPLC-ESI-MS/MS) has been widely used as a powerful means for the analysis of multi-components in TCMs. Recently, with the development of various data acquisition methods, high-resolution mass spectrometry (HRMS), especially linear ion trap-Orbitrap mass spectrometer (LTQ-Orbitrap MS), has exhibited excellent performances in constituents detection owing to its high-speed and detection sensitivity [2]. UHPLC provides effective chromatographic separation, while LTQ-Orbitrap provides multi-stage mass spectra using data-dependent analysis with a higher mass resolution and mass accuracy than many other mass spectrometers [2]. Therefore, UHPLC-ESI-MS/MS remarkably facilitates the identification of known and unknown components in TCM extracts with high sensitivity and accuracy [3,4,5].

Menthae Haplocalycis herba, one popular TCM, derived from the aerial part (stem and leaf) of *Menthae haplocalyx* Briq., is commonly used for the treatment of wind-heat cold, pharyngitis, measles, rubella in dispelling wind and heat. It has been officially documented in Chinese Pharmacopoeia (Version 2015) named “Bo He” and used as a Chinese edible herb [6]. In the past few decades, systematic chemical and pharmacological studies have been performed mainly on the volatile constituents [7,8]. However, studies in recent years have suggested that volatile constituents of Menthae Haplocalycis herba can lead to a series of toxic effects, such as liver injury and other toxic symptoms [9,10]. It is well known that Menthae Haplocalycis herba is clinically used as decoction, which indicates that non-volatile components should be responsible for its efficacy. For instance, flavonoids, phenolic acids and some terpenoids from Menthae Haplocalycis herba show various activities of anti-viral, anti-inflammatory and anti-oxidation, which is probably closely related to its traditional efficacy [11,12]. Limited knowledge about the non-volatile constituents hinders its application in clinical practice and retards its modernization process. Hence, it will be of great importance to characterize the non-volatile constituents in Menthae Haplocalycis herba.

Herein, a rapid and sensitive UHPLC-ESI-MS/MS method was established to systematically profile the non-volatile constituents in Menthae Haplocalycis herba, which may contribute to new drug development and effective substance basis clarification.

## 2. Results and Discussion

**Figure 1:**
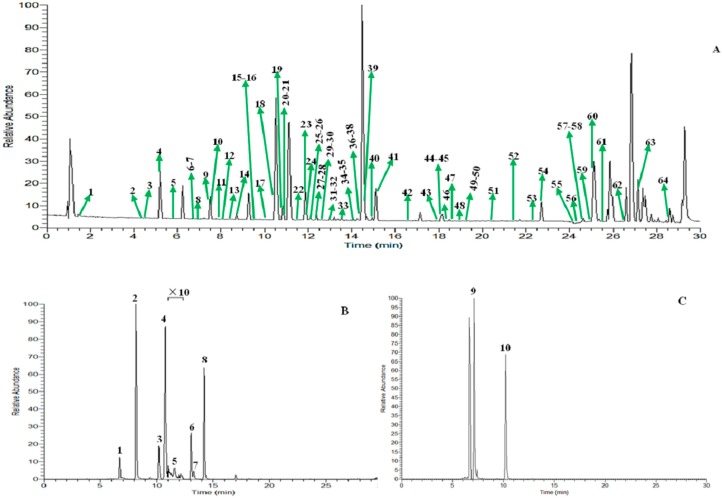
(**A**) Base peak chromatogram of Menthae Haplocalycis herba in positive ion mode; (**B**) base peak chromatogram of mixed reference solution of Menthae Haplocalycis herba. (**1**. Bohecineole A, **2**. Luteolin-7-*O*-glucoside, **3**. Diosmin, **4**. Hesperidin, **5**. Rosmarinic acid, **6**. Lithospermic Acid, **7**. Salvianolic acid B, **8**. Buddleoside); (**C**) base peak chromatogram of mixed reference solution of Menthae Haplocalycis herba. (**9**. (1*R*,2*R*,4*S*)-*trans*-1,8-cineole-2-*O*-*β*-d-glucopyranoside, **10**. Naringin). “×10” magnified ten-fold.

**Figure 2:**
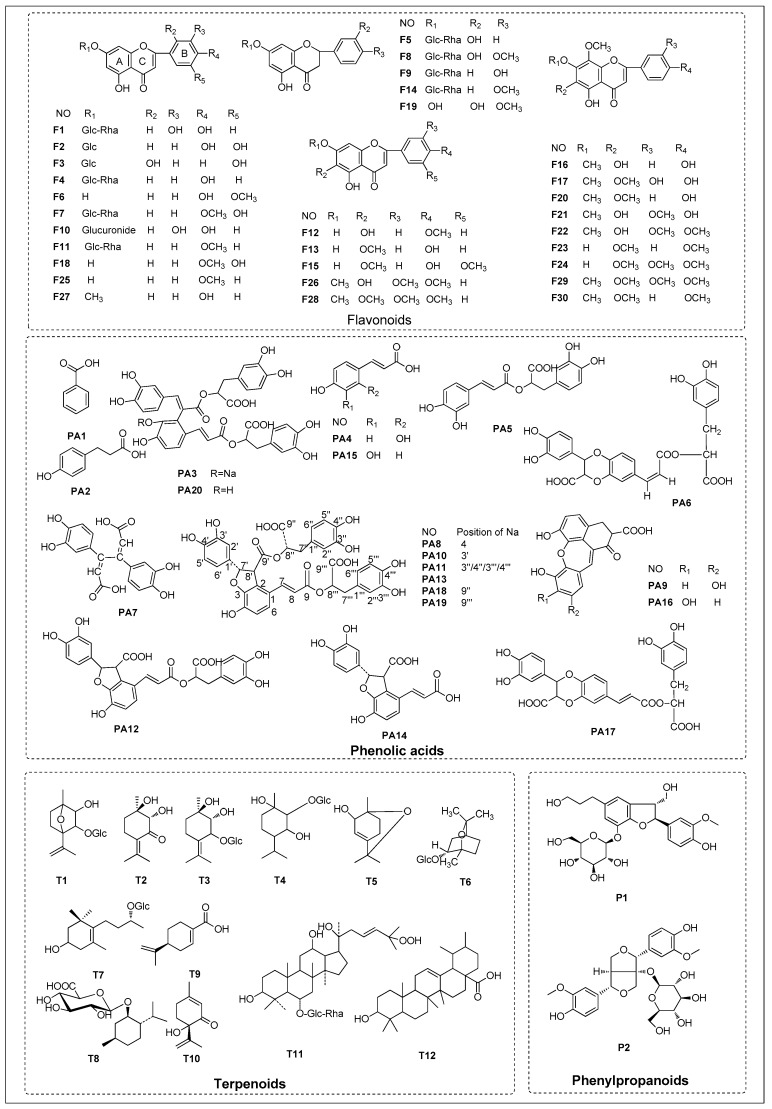
The structures of chemical constituents identified in Menthae Haplocalycis herba by UHPLC-ESI-MS/MS.

**Figure 3:**
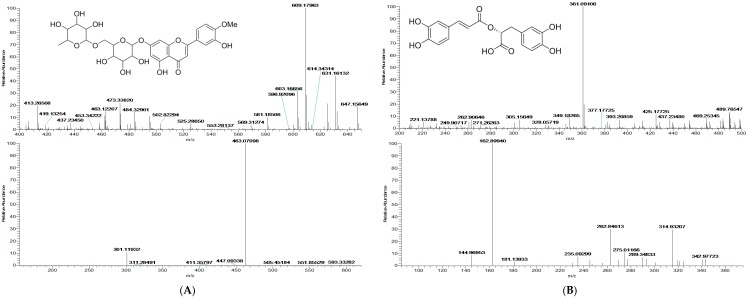

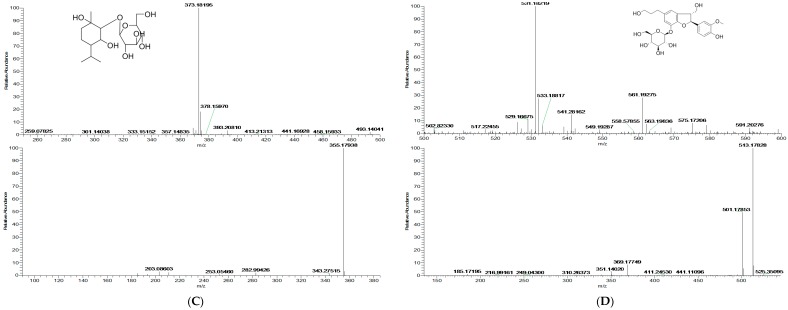
The representative mass spectra of chemical constituents identified in Menthae Haplocalycis herba in positive ion mode of Diosmin (**A**); Rosmarinic acid (**B**); Bohenoside A (**C**) and Clemastanin A (**D**).

Positive ion mode was employed for the comprehensive analysis and the base peak chromatograms (BPC) are shown in Figure 1. Based on MS spectra and retention time (t_R_), a total of 64 compounds (Table 1, Figure 2) were unambiguously identified or tentatively characterized. Among them, 10 constituents were positively identified by comparing retention times and MS data with respective reference compounds. The representative mass spectra of chemical constituents identified in Menthae Haplocalycis herba in positive ion mode were displayed in Figure 3 as well.

### 2.1. Characterization of Flavonoids 

The developed UHPLC-ESI-MS/MS method is effective to obtain information on the carbohydrate sequence and aglycone moiety. Cleavage at the glycosidic *O*-linkages with a concomitant H-rearrangement leads to the elimination of monosaccharide residues, i.e. the loss of 146 u (deoxyhexose), 162 u (hexose) or 176 u (uronic acid), allowing the determination of carbohydrate sequence. Moreover, the fragment ions produced by Retro–Diels–Alder (RDA) reactions are useful in terms of flavonoid aglycone identification since they can provide information on the number and type of substituents on A-and B-rings [13]. Finally, 30 flavonoids were identified, including five flavanones (two flavanone aglycones and three flavanone glycosides) and 25 flavonoids (18 flavonoid aglycones and seven flavonoid glycosides).

#### 2.1.1. Structural Characterization of Flavanones

Compounds **F9** and **F19** produced [M + H]^+^ ions at *m*/*z* 303.08572 (C_16_H_15_O_6_, error < 5 ppm). In the MS/MS spectra, they generated the similar fragment ions at *m*/*z* 117 corresponding to loss of B-ring from [M + H]^+^ ion, which indicated they might be flavonones. The fragment ions at *m*/*z* 153, *m*/*z* 151 and *m*/*z* 137 were yielded by RDA cleavage, indicating there were two -OH on A-ring and one -OH and one -OCH_3_ on B-ring. Combining the literature data and Clog *P* values, **F9** and **F19** were identified as Homoeriodictyol and Hesperetin, respectively [14,15]. 

Compound **F8** showed [M + H]^+^ ion at *m*/*z* 611.19635 (C_28_H_35_O_15_, error < 5 ppm). The major fragment ions at *m*/*z* 465 and *m*/*z* 303 were yielded by successive neutral loss of Rha (146 Da) and Glc (162 Da) from [M + H]^+^ ions. By comparing retention time, fragmentation behaviors with reference substance, it was unambiguously identified as Hesperidin [15,16]. 

Compounds **F5** and **F14** yielded their respective [M + H]^+^ ions at *m*/*z* 581.18488 (C_27_H_33_O_14_) and *m*/*z* 595.20213 (C_28_H_35_O_14_) with mass errors within 5 ppm. Both of them generated [M + H − H_2_O]^+^, [M + H − Rha]^+^, [M + H − Glc]^+^ and [M + H − Rha − Glc]^+^ ions at *m*/*z* 563, *m*/*z* 577; *m*/*z* 435, *m*/*z* 449; *m*/*z* 419, *m*/*z* 433; *m*/*z* 273, *m*/*z* 287, respectively. Finally, **F5** was unambiguously identified as Didymin by comparing with the authentic compound, while **F14** was tentatively deduced as Naringin by analyzing the fragment ions produced by RDA cleavage [15,16,17].

#### 2.1.2. Structural Characterization of Flavonoids

Compound **F6**, **F13** and **F18** showed [M + H]^+^ ions at *m*/*z* 301.07066 (C_16_H_13_O_6_, error < 5 ppm). The fragment ions yielded by RDA cleavage demonstrated that their substituent positions were remarkably different. By referring to the literature data, they were respectively identified as Chrysoeriol, Hispidulin and Diosmetin [16,18,19,20]. Likewise, compounds **F12**, **F25**, **F27**; **F15**, **F16**; **F17**, **F21**; **F20**, **F23**, **F26**; **F22**, **F24** and **F28**, **F30** were finally identified as 6,7-Dihydroxy-4’-methoxyisoflavone, Acacetin and Genkwanin [16,17]; Jaceosidin, 5,6,4’-Trihydroxyl-7,8-dimethoxy flavone [21]; Sideritiflavone, Thymonin [22]; Xanthomicrol, Nevadensin, 5,6-Dihydroxy-7,3’,4’-trimethoxy flavone [17,23]; 5,6-Dihydroxy-7,8,3’,4’-tetrame-thoxyflavone, 5,7-Dihydroxy-6,8,3’,4’-tetramethoxyflavone [16]; 5-Hydroxy-6,7,3’,4’-tetramethoxyflavones, Gardenin B [17,24], respectively. 

Compound **F29** generated [M + H]^+^ ion at *m*/*z* 389.12309 (C_20_H_21_O_8_, error < 5 ppm). Once reaching CID (collision-induced dissociation) mode, it further underwent a series of methyl units losing and RDA cleavage, which generated product ions at *m*/*z* 374, *m*/*z* 359, *m*/*z* 227 and *m*/*z* 165. Therefore, **F29** was tentatively identified as 5-Hydroxy-6,7,8,3’,4’-pentamethoxyflavone.

Compound **F1** gave [M + H]^+^ ion at *m*/*z* 595.16574 (C_27_H_31_O_15_, error < 5 ppm). The major ions at *m*/*z* 449 and *m*/*z* 287 in its ESI-MS^2^ spectrum indicated the presence of rutinoside. By analyzing fragment ions and comparing with literature data, **F1** was plausibly defined as Luteolin-7-*O*-rutinoside [16].

Compounds **F3** and **F10** produced [M + H]^+^ ions at *m*/*z* 463.01780 (C_21_H_19_O_12_, error < 5 ppm). After the CID cleavage, their further fragmentation all resulted in [M + H − H_2_O]^+^ ion at *m*/*z* 445, [M + H − 2H_2_O]^+^ ion at *m*/*z* 427 and [M + H − Glucuronic acid]^+^ ion at *m*/*z* 287. According to the fragmentation pathways and Clog *P* values, **F3** and **F10** were tentatively identified as 2-(2,5-Dihydroxyphenyl)-5-hydroxy-4-oxo-4*H*-chromen-7-yl-*β*-d-glucopyranosiduronic acid and Luteolin-7-*O*-glucuronide, respectively.

Compound **F7** exhibited [M + H]^+^ ion at *m*/*z* 609.17999 (C_28_H_33_O_15_, error < 5 ppm). Its ESI-MS^2^ spectrum gave the fragment ions at *m*/*z* 463 and *m*/*z* 301, involving successive loss of rhamnosyl and glucosyl groups. The ion at *m*/*z* 301 further generated predominant fragment ions at *m*/*z* 286 and *m*/*z* 258, which were similar to Diosmetin [15,16,18,25]. The deduced result was further confirmed by comparing with an authentic compound.

Similarly, based on literature data or reference compounds, compounds **F2**, **F4**, and **F11** were identified as Luteolin-7-*O*-glucoside, Apigenin-7-*O*-rutinoside and Buddleoside, respectively [15,16,17,18].

### 2.2. Characterization of Phenolic Compounds

In the preliminary study, we found that UV absorption spectra of some peaks in the fingerprints of Menthae Haplocalycis herba were similar to salvianolic acid compounds. The basic structure units of salvianolic acid are tanshinol and caffeic acid. Thus, in their ESI-MS/MS spectra, regular fragment ions including [M + H − C_9_H_8_O_4_]^+^ and [M + H − C_9_H_10_O_5_]^+^ are produced by the neutral loss of caffeic acid and tanshinol. Moreover, owing to the existence of carboxyl and carbonyl, it is common to observe the neutral loss of CO and CO_2_. According to these fragmentation patterns, 20 phenolic acids were positively or tentatively identified.

Compound **PA2** produced [M + H]^+^ ion at *m*/*z* 167.07027 (C_9_H_11_O_3_, error < 5 ppm). It firstly produced the ESI-MS^2^ base peak ion at *m*/*z* 149 by losing H_2_O. Upon reaching CID mode, [M + H]^+^ ion further generated [M + H − CO]^+^, [M + H − H_2_O − CO]^+^ and C_7_H_7_O^+^ ions at *m*/*z* 139, *m*/*z* 121 and *m*/*z* 107, respectively. According to the above analysis, **PA2** was tentatively defined as Phloretic acid. Similarly, based on the above analysis, **PA1** was tentatively identified as Benzoic acid.

Compounds **PA4** and **PA15** exhibited the same [M + H]^+^ ions at *m*/*z* 181.04953 (C_9_H_9_O_4_, error < 5 ppm). Both of them firstly generated ESI-MS^2^ base peak ions at *m*/*z* 163 by loss of H_2_O. The major fragment ions in the ESI-MS^2^ spectra were *m*/*z* 163, *m*/*z* 153, *m*/*z* 135 and *m*/*z* 117, suggesting the presence of -COOH. Compared with literature data and Clog *P* values, **PA4** and **PA15** were plausibly characterized as 2,4-Dihydroxycinnamic acid and Caffeic acid, respectively [26]. Meanwhile, by comparing with authentic standards and literature, **PA5** and **PA13** were identified as Rosmarinic acid and Salvianolic acid B, respectively [12,27,28].

Compounds **PA6**, **PA12** and **PA17** showed [M + H]^+^ ions at *m*/*z* 539.11840 (C_27_H_23_O_12_, error < 5 ppm). After the CID cleavage, the further fragmentation of *m*/*z* 539 resulted in [M + H − C_9_H_10_O_5_]^+^ at *m*/*z* 341 and [M + H − C_9_H_10_O_5_ − CO_2_]^+^ at *m*/*z* 297, involving the presence of tanshinol. By referring to literature data, the properties of *Cis*-salvianolic acid J, Lithospermic acid and *trans*-salvianolic acid J were in accordance with the description. Therefore, **PA12** was unambiguously defined as Didymin by comparing with reference compound, while **PA6** and **PA17** were finally deduced as *Cis*-salvianolic acid J and *trans*-salvianolic acid J according to the literature data and Clog *p* values [12,26,27,29]. 

Compounds **PA7** and **PA14** yielded [M + H]^+^ ions at *m*/*z* 359.07614 (C_18_H_15_O_8_, error < 5 ppm). The major fragment ions in their ESI-MS/MS spectra were *m*/*z* 341 [M + H − H_2_O]^+^, *m*/*z* 313 [M + H − H_2_O − CO]^+^ and *m*/*z* 179 [M + H − C_9_H_8_O_4_]^+^, indicating the presence of caffeic acid. By referring to the literature data and Clog *p* values, **PA7** and **PA14** were tentatively defined as Bis (3,4-dihydroxybenzylidene) succinic acid and Prolithospermic acid, respectively [26]. 

Compounds **PA9** and **PA16** displayed [M + H]^+^ ions at *m*/*z* 341.06557 (C_18_H_13_O_7_, error < 5 ppm). Both of their [M + H]^+^ ions generated a series of fragment ions at *m*/*z* 323 [M + H − H_2_O]^+^, *m*/*z* 297 [M + H − CO_2_]^+^, *m*/*z* 295 [M + H − H_2_O − CO]^+^, *m*/*z* 279 [M + H − CO_2_ − H_2_O]^+^ and *m*/*z* 267 [M + H − H_2_O − 2CO]^+^. According to the literature data and Clog *P* values, **PA9** and **PA16** were tentatively identified as Benzo[*f*] naphthol [1,8-*bc*] oxepine-8-carboxylic acid and Salvianolic acid G, respectively [29].

Compound **PA20** showed [M + H]^+^ ion at *m*/*z* 719.16066 (C_36_H_31_O_16_, error < 5 ppm). The fragment ions observed at *m*/*z* 539 [M + H − C_9_H_8_O_4_]^+^, *m*/*z* 493 [M + H − C_9_H_10_O_5_ − CO]^+^ and *m*/*z* 297 [M + H − C_9_H_8_O_4_ − C_9_H_10_O_5_ − CO_2_]^+^ in the ESI-MS^2^ spectrum indicated the presence of caffeic acid and tanshinol. By comparison with the literature data, **PA20** was finally identified as Salvianolic acid E [29]. Likewise, **PA3**, **PA8**, **PA10**, **PA11**, **PA18** and **PA19** all produced the identical [M + H]^+^ ions at *m*/*z* 741.14260 (C_36_H_30_NaO_16_, error < 5 ppm). They all have the similar fragmentation pathways and characteristic fragment ions. According to their Clog *P* values, they were tentatively identified and differentiated. As a result, the sodium was monitored at C-3, C-4, C-3’, C-3’’/4’’/3’’’/4’’’, C-9’’ and C-9’’’, respectively. By comparison with the bibliography and ESI-MS^2^ fragmentation data, **PA3** was deduced as Sodium salvianolic acid E while **PA8**, **PA10**, **PA11**, **PA18** and **PA19** were tentatively assigned as and Sodium lithospermate B, respectively [29]. 

### 2.3. Characterization of Terpenoids

It is difficult to determine terpenoids by UHPLC-PDA analysis because of their weak UV absorption. UHPLC-MS/MS is a powerful technique to identify these kinds of constituents. In their ESI-MS/MS spectra, terpenoids usually lose a molecule of H_2_O or CH_3_ because it normally contains hydroxy and methyl groups. For terpenoids glycosides, [M + H − 162]^+^ was easily monitored as the characteristic ion by losing a dehydrated glucose. Moreover, fragment ions referred above often have high abundance. Based on these fragmentation pathways, four monoterpenoid aglycones, six monoterpenoids glycosides and two triterpenoids were finally identified.

#### 2.3.1. Identification of Monoterpenoid Aglycones

Compound **T2** gave [M + H]^+^ ion at *m*/*z* 185.11722 (C_10_H_17_O_3_, error < 5 ppm). Upon CID mode, it further generated [M + H − H_2_O]^+^, [M + H − CO]^+^, [M + H − 2H_2_O]^+^ and [M + H − H_2_O − CO]^+^ ions at *m*/*z* 167, *m*/*z* 157, *m*/*z* 149 and *m*/*z* 139, respectively. According to the above analysis, **T2** was tentatively identified as (1R*,2S*)-1,2-dihydroxy-*ρ*-menth-4(8)-en-3-one. Similarly, based on the above analysis and authentic compound, **T5** was unambiguously identified as Bohecineole A [30].

Compounds **T9** and **T10** were a pair of isomers. Both of them gave the identical [M + H]^+^ ions at *m*/*z* 167.10665 (C_10_H_15_O_2_, error < 5 ppm) and similiar ESI-MS/MS fragment ions at *m*/*z* 149, *m*/*z* 139, *m*/*z* 125, *m*/*z* 121, *m*/*z* 95 and *m*/*z* 93. Nevertheless, their ion abundances were remarkably different. The ion at *m*/*z* 149 was yielded by neutral loss of H_2_O from [M + H]^+^ ion and further produced fragment ion at *m*/*z* 121 by losing carbonyl. According to these fragmentation pathways, **T9** and **T10** were tentatively identified as (*S*)-(−)-Perillic acid and (4S*)-4-hydroxy-*ρ*-mentha-1,8-dien-3-one, respectively.

#### 2.3.2. Identification of Monoterpenoid Glycosides

With respect to compound **T1**, [M + Na]^+^ adduct ion at *m*/*z* 369.15198 (C_16_H_26_O_8_Na, error < 5 ppm). After the CID cleavage, *m*/*z* 351, *m*/*z* 328, *m*/*z* 207 and *m*/*z* 185 were produced by successively neutral loss of one molecular of water and dehydrated glucose. According to the above analysis, **T1** was plausibly described as petroside [31]. Likewise, by comparing with the authentic compound, **T6** was unambiguously identified as (1*R*,2*R*,4*S*)-*trans*-1,8-cineole-2-*O*-*β*-d-glucopyranoside.

Similarly, based on the above analysis, **T3**, **T4**, **T7** and **T8** were tentatively deduced as (2*R*,3*R*,4*S*,5*S*,6*R*)-2-(((1*S*,2*R*,3*S*)-2,3-dihydroxy-3-methyl-6-(propan-2-ylidene)cyclohexyl)oxy)-6-(hydroxymethyl)tetrahydro-2*H*-pyran-3,4,5-triol, Bohenoside A, Linarionoside B and (1*R*,2*S*,5*R*)-(−)-methol *β*-d-Glucuronide, respectively. 

#### 2.3.3. Identification of Triterpenoids

Compounds **T11** and **T12** produced their respective [M + H]^+^ ions at *m*/*z* 817.49439 (C_42_H_73_O_15_) and 457.36762 (C_30_H_49_O_3_) with mass errors within 5 ppm. The [M + H]^+^ ion of **T11** generated [M + H − H_2_O]^+^ ion at *m*/*z* 799, [M + H − H_2_O − CO]^+^ ion at *m*/*z* 771 and [M + H − Glc]^+^ ion at *m*/*z* 655, etc. By comparing with the literature data, **T11** was tentatively identified as Floralquinquenoside C [32]. Upon CID mode, **T12** further generated a series of fragment ions at *m*/*z* 439, *m*/*z* 411, *m*/*z* 393 and *m*/*z* 191. The ion at *m*/*z* 439 was yielded by neutral loss of H_2_O from [M + H]^+^ ion and then produced fragment ions at *m*/*z* 191 by RDA cleavage. By comparing with the literature data, **T12** was finally identified as Ursolic acid [33].

Our data demonstrated that some monoterpenoids are the glycosides of volatile constiuents in Menthae Haplocalycis herba. Taking **T8** for example, it is the glucuronide of menthol, as we all know that menthol is the main effective component attributed to volatile constituents in Menthae Haplocalycis herba. Meanwhile, some monoterpenoid glycosides may be metabolized by intestinal flora after hydrolysis of aglycones (probably volatile constituents) and then absorbed into blood to display pharmacological effects. In this sense, it will be of great significance to carry out the study of non-volatile constituents in Menthae Haplocalycis herba.

### 2.4. Characterization of Phenylpropanoids

In the ESI-MS/MS spectra, phenylpropanoids always lose a molecule of H_2_O because they contain hydroxy groups. For phenylpropanoids glycosides, [M + H − 162]^+^ was easily monitored as their characteristic fragment ion. According to the fragmentation pathways, two phenylpropanoids glycosides were tentatively identified.

Compounds **P1** and **P2** generated their respective [M + Na]^+^ adduct ions at *m*/*z* 531.18368 (C_25_H_32_O_11_Na) and *m*/*z* 559.17859 (C_26_H_32_O_12_Na) with mass errors within 5 ppm. Both of them yielded [M + Na − H_2_O]^+^ and [M + Na − Glc]^+^ ions at *m*/*z* 513, *m*/*z* 369 and *m*/*z* 541, *m*/*z* 397, respectively. Thus, **P1** and **P2** were tentatively determined as Clemastanin A and (+)-1-Hydroxypinoresinol-1-*O*-*β*-d-glucoside, respectively.

### 2.5. Target Isolation and Verification

Acacetin, Rosmarinic acid and Clemastanin A were obtained from the effluent fraction of H_2_O–MeOH (50:50⟶75:25, *v*/*v*) by multiple isolation means. Their structures were verified combined with the ^1^H-NMR, ^13^C-NMR, which consistent with bibliographies [34,35,36,37]. The obtained MS data [35,36,37] of them were in accordance with the deduction of the MS/MS results, which further demonstrated the reliability of the deduced fragmentation patterns and identified the profile of non-volatile constituents in Menthae Haplocalycis herba.

## 3. Materials and Methods

### 3.1. Materials and Reagents

HPLC grade acetonitrile and formic acid were supplied by Fisher Scientific (Fisher, Fair Lawn, NJ, USA). Ultrapure water was purchased from Hangzhou Wahaha Group Co., Ltd. (Hangzhou, Zhejiang, China). All of the other reagents and chemicals were of analytical grade and commercially available.

Reference compounds including Luteolin-7-*O*-glucoside, Lithospermic acid, Bohecineole A, (1*R*,2*R*,4*S*)-*trans*-1,8-cineole-2-*O*-*β*-d-glucopyranoside and Salvianolic acid B were prepared from Menthae Haplocalycis herba by authors. Their structures were fully characterized by chemical and spectroscopic methods (UV, IR, NMR and MS) [38,39]. Hesperidin and Rosmarinic acid were purchased from National Institutes for Food and Drug Control (Beijing, China). Buddleoside, Diosmin and Naringin were purchased from Chengdu Must Bio-Technology Co., Ltd. (Chengdu, Sichuan, China). All of these reference compounds showed purities of above 98% by HPLC analysis. 

Dried Herbal medicine samples of Menthae Haplocalycis herba were purchased from Anguo Linshi Medicinal Materials Co., Ltd. in Hebei, China and were authenticated as the aerial part of *Menthae. haplocalyx* Briq, which was harvested in Jiangsu at autumn by Professor Chunsheng Liu at the Beijing University of Chinese Medicine (BUCM, Beijing, China). All Menthae Haplocalycis herba samples were stored in Chinese medicine institutes of BUCM.

### 3.2. Sample Preparation

#### 3.2.1. Standard Solutions

Stock solutions were prepared by dissolving appropriate amounts of 10 reference compounds in methanol. Proper amounts of each stock solution were then transferred to a 25 mL volumetric flask, and then methanol was added to make up the volume to obtain a final mixed reference solution. All the solutions were stored at 4 °C and brought to room temperature before use.

#### 3.2.2. Sample Solutions

Sample (0.2 g) milled by 65 meshes beforehand were extracted with 10 mL methanol in an ultrasonic bath for 30 min. After being cooled to room temperature, it was weighed and adjusted to the original weight by adding methanol, and then filtered through a 0.22 μm nylon filter for analysis.

### 3.3. UHPLC-ESI-MS/MS System

UHPLC-ESI-MS/MS analysis was performed on a DIONEX Ultimate 3000 UHPLC system (Thermo Fisher Scientific, Waltham, MA, USA) with a binary pump and an autosampler. A series of preliminary experiments were performed to optimize mobile phase composition and elution conditions. Finally, analysis was carried out at 35 °C on an Acquity UPLC^®^ BEH C_18_ column (2.1 mm × 100 mm, 1.7 μm, Waters Corporation, Milford, MA, USA). The mobile phase consisted of 0.2% formic acid aqueous solution (A) and acetonitrile (B). A gradient program was adopted as follows: 0–5 min, 5%–19.5% B; 5–8.5 min, 19.5% B; 8.5–11 min, 19.5–27% B; 11–15 min, 27% B; 15–22 min, 27–40% B; 22–24 min, 40–55% B; 24–26 min, 55–75% B; 26–28.5 min, 75% B; 28.5–30% min, 75–100% B. The flow rate was kept at 0.30 mL/min and the sample volume injected was 2 μL.

The optimized operating parameters in positive ion mode were listed as follows: capillary temperature of 350 °C; sheath gas flow rate of 40.0 arb; auxiliary gas flow rate of 20.0 arb; source voltage of 4 kV; capillary voltage of 25 V, and tube lense of 110 V. HRMS analysis was operated with a mass range of *m*/*z* 100–1000 at a resolving power of 30,000.

### 3.4. Peak Selections and Data Processing

Thermo Xcalibur 2.1 workstation (Thermo Fisher Scientific, San Jose, CA, USA) was used for data acquisition and processing. In order to obtain as many fragment ions of non-volatile compounds of Menthae Haplocalycis herba as possible, the peaks detected with intensity over 30,000 were selected for identification. The chemical formulas for all parent ions of the selected peaks were calculated from the accurate mass using a formula predictor by setting the parameters as follows: C (0–50), H (0–100), O (0–30), Cl (0–2), N (0–2), Na (0–2), K (0–1) and ring double bond (RDB) equivalent value (0–20). Other elements such as Br and P were not considered because they are rarely present in Menthae Haplocalycis herba.

### 3.5. Extraction and Isolation of Target Compounds

The air dried Menthae Haplocalycis herba samples (20.0 Kg) were extracted three times with tenfold excess of 70% EtOH under reflux for 1.5 h each at 80 °C. The combined extract was evaporated under reduced pressure to obtain a crude residue. This residue was further dispersed in H_2_O, and then successively passed through a Dianion HP (Mitsubishi Chemical Co., Kyoto, Japan) 2MGL macroporous resin column and then washed with extracted with H_2_O–MeOH (5:95⟶MeOH, *v*/*v*). The H_2_O–MeOH (50:50⟶75:25, *v*/*v*) extract was further purified by multiple isolation methods, such as silica gel column chromatography, C_18_ antiphase silica gel column chromatography, Sephadex LH-20 gel chromatography, HPD-400 macroporous resin column, etc. Acacetin, Rosmarinic acid and Clemastanin A were obtained finally. 

## 4. Conclusions

Our study took advantage of the UHPLC-LTQ-Orbitrap HRMS system and firstly reported the identification of 64 non-volatile compounds with various structure types, including 30 flavonoids, 20 phenolic acids, 12 terpenoids and two phenylpropanoids in Menthae Haplocalycis herba. Finally, target isolation of three compounds named Acacetin, Rosmarinic acid and Clemastanin A were performed based on the obtained results, which further confirmed the deduced fragmentation patterns and identified the profile of non-volatile constituents in Menthae Haplocalycis herba. The results also clearly elucidated that there may exist some inevitable relations between volatile and non-volatile constituents. Meanwhile, our developed method has been shown to be an excellent tool for the systematic characterization of non-volatile constituents in Menthae Haplocalycis herba, which also benefits its further pharmacological research and clinical applications. Moreover, this study sets a good example for the rapid identification of chemical constituents in TCMs.

## Figures and Tables

**Table 1:** Summary of chemical constituents identified in Menthae Haplocalycis herba by UHPLC-ESI-MS/MS.

Peak		*t*_R_ (min)	Compound Formula	Identification	Experimental Mass *m*/*z*	Theoretical Mass *m*/*z*	Mass Error (× 10^−6^)	MS^2^ Data(Measured)
**1**	**PA1**	1.52	C_7_H_7_O_2_	Benzoic acid	123.04371	123.04405	−2.81	95(100),82(2),81(16),67(3),57(2)
**2**	**T1**	4.41	C_16_H_26_O_8_Na ^b^	Petroside	369.15112	369.15198	−2.35	352(7),351(47),328(27),307(12),285(9),207(100),203(34),185(22),149(6)
**3**	**T2**	4.63	C_10_H_17_O_3_	(1R*,2S*)-1,2-dihydroxy-*ρ*-menth-4(8)-en-3-one	185.11687	185.11722	−1.89	168(4),167(100),157(6),149(18),139(19),125(32),121(26)
**4**	**T3**	5.21	C_16_H_28_O_8_Na ^b^	(2*R*,3*R*,4*S*,5*S*,6*R*)-2-(((1*S*,2*R*,3*S*)-2,3-dihydroxy-3-methyl-6-(propan-2-ylidene)cyclohexyl)oxy)-6-(hydroxymethyl)tetrahydro-2*H*-pyran-3,4,5-triol	371.16702	371.16763	−1.66	355(2),353(0.2),203(100),201(6),193(4),191(2)
**5**	**T4**	5.82	C_16_H_30_O_8_Na ^b^	Bohenoside A	373.18250	373.18328	−2.11	357(11),356(10),355(100),346(4),211(4)
**6**	**T5**	6.71	C_10_H_17_O_2_	Bohecineole A ^a^	169.12190	169.12230	−2.40	151(71),133(7),123(86),113(5),109(56),107(100),58(4) ^△^
		6.75	C_10_H_16_O_2_Na ^b^		191.10388	191.10425	−1.94	
**7**	**P1**	6.71	C_25_H_32_O_11_Na ^b^	Clemastanin A	531.18329	531.18368	−0.74	514(10),513(100),502(4),501(46),369(9),351(3)
**8**	**PA2**	7.01	C_9_H_11_O_3_	Phloretic acid	167.06995	167.07027	−1.92	149(100),139(23),126(42),125(57),121(40),109(10),107(49),97(10)
**9**	**T6**	7.11	C_16_H_28_O_7_Na ^b^	(1*R*,2*R*,4*S*)-*trans*-1,8-cineole-2-*O*-*β*-d-glucopyranoside ^a^	355.17206	355.17272	−1.87	337(14),285(100),268(53),193(6),185(9),135(2) ^△^
		7.30			355.17209		−1.78	337(12),285(3),267(36),257(2),203(21),201(100),193(4),185(2)
**10**	**P2**	7.62	C_26_H_32_O_12_Na ^b^	(+)-1-Hydroxypinoresinol-1-*O*-*β-*d-glucoside	559.17682	559.17859	−3.17	541(19),536(4),515(28),437(7),398(9),397(100),396(15),395(65),337(67),309(24)
**11**	**F1**	7.95	C_27_H_31_O_15_	Luteolin-7-*O*-rutinoside	595.16522	595.16574	−0.88	577(0.3),549(0.4),449(100),287(12)
**12**	**F2**	8.19	C_21_H_21_O_11_	Luteolin-7-*O*-glucoside ^a^	449.10611	449.10783	−3.84	287(100) ^△^
		8.16			449.10635		−3.31	431(3),416(1),287(100),269(2)
**13**	**F3**	8.45	C_21_H_19_O_12_	2-(2,5-Dihydroxyphenyl)-5-hydroxy-4-oxo-4*H*-chromen-7-yl *β*-d-glucopyranosiduronic acid	463.08578	463.08710	−2.85	445(0.3),427(0.2),375(0.2),287(100)
**14**	**T7**	8.75	C_19_H_34_O_7_Na ^b^	Linarionoside B	397.21912	397.21967	−1.39	379(100),326(28),217(26),203(88),187(30)
**15**	**F4**	9.48	C_27_H_31_O_14_	Apigenin-7-*O*-rutinoside	579.16974	579.17083	−1.88	561(1),433(100),417(1),271(9)
**16**	**F5**	10.23	C_27_H_33_O_14_	Naringin ^a^	581.18488	581.18648	−2.75	563(59),545(50),527(30),509(11),435(99),419(100),417(92),401(15),383(9),315(27),273(30) ^△^
		9.58			581.18573		−1.29	563(20),545(12),527(7),419(100),273(24)
**17**	**F6**	10.19	C_16_H_13_O_6_	Chrysoeriol	301.07007	301.07066	−1.97	286(6),286(100),270(0.49),255(3),183(0.5),121(0.23)
**18**	**F7**	10.35	C_28_H_33_O_15_	Diosmin ^a^	609.17963	609.18139	−2.90	591(1),573(0.2),463(100),447(1),301(11) ^△^
		10.50			609.17999		−2.30	591(0.1),463(100),447(1),429(0.3),301(11),286(0.1),258
**19**	**PA3**	10.64	C_36_H_30_O_16_Na	Sodium salvianolic acid E	741.14203	741.14260	−0.77	723(1),561(100),543(28),381(3),363(4),345(2),317(2)
**20**	**F8**	10.77	C_28_H_35_O_15_	Hesperidin ^a^	611.19617	611.19704	−1.43	593(36),575(34),491(12),489(25),465(97),449(100),447(75),431(26),345(30),303(45) ^△^
		10.92			611.19635		−1.14	593(19),575(33),557(14),491(10),489(21),465(62),449(100),447(59),431(22),345(17),303(42)
**21**	**F9**	10.98	C_16_H_15_O_6_	Homoeriodictyol	303.08563	303.08630	−2.25	285(13),179(46),177(100),153(28),151(5),136(1)
**22**	**PA4**	11.53	C_9_H_9_O_4_	2,4-Dihydroxycinnamic acid	181.04900	181.04953	−2.95	163(100),153(0.4),139(3),137(0.2),135(2),121(0.1),117(0.1)
**23**	**PA5**	11.56	C_18_H_17_O_8_	Rosmarinic acid ^a^	361.09134	361.09179	−1.25	347(10),343(24),333(7),328(12),293(11),263(9),191(13),164(5),163(100),145(7) ^△^
		11.84			361.09122		−1.58	
**24**	**F10**	11.94	C_21_H_19_O_12_	Luteolin-7-*O*-glucuronide	463.08563	463.08710	−3.17	445(0.5),427(0.4),405(0.3),288(1),287(100)
**25**	**PA6**	12.14	C_27_H_23_O_12_	*Cis*-salvianolic acid J	539.11725	539.11840	−2.13	539(7),521(7),495(6),493(6),393(5),341(100),323(3),297(8),295(9),179(5)
**26**	**PA7**	12.28	C_18_H_15_O_8_	Bis(3,4-dihydroxybenzylidene)succinic acid	359.07587	359.07614	−0.76	341(100),323(3),313(34),297(4),295(10),285(7),221(15),179(1),123(16)
**27**	**PA8**	12.28	C_36_H_30_O_16_Na	Sodium lithospermate B	741.14203	741.14260	−0.77	723(0.3),579(100),561(25),533(19),517(19),381(4),355(3)
**28**	**PA9**	12.39	C_18_H_13_O_7_	2,4,12-Trihydroxy-7-oxo-8,9-dihydro-7*H*-benzo[*f*]naphtho[1,8-*bc*]oxepine-8-carboxylic acid	341.06497	341.06557	−1.78	323(96),313(18),297(63),295(100),279(18),277(22),267(2),253(12),249(18)
**29**	**PA10**	12.61	C_36_H_30_O_16_Na	Sodium lithospermate B	741.14233	741.14260	−0.37	579(100),561(27),533(25),517(20),399(5),355(3)
**30**	**T8**	12.65	C_16_H_28_O_7_Na ^b^	(1*R*,2*S*,5*R*)-(−)-methol *β*-d-Glucuronide	355.17181	355.17272	−2.57	340(7),339(3),337(11),325(5),323(13),309(4),295(6),285(13),267(100),257(16),205(6),183(3)
**31**	**PA11**	13.04	C_36_H_30_O_16_Na	Sodium lithospermate B	741.14197	741.14260	−0.85	561(100),543(59),517(23),363(11),362(19),319(3)
**32**	**PA12**	13.06	C_27_H_23_O_12_	Lithospermic acid ^a^	539.11725	539.11840	−2.13	539(31),538(28),521(100),493(19),481(15),452(18),393(23),231(21),199(21) ^△^
		13.07	C_27_H_23_O_12_		539.11768		−1.34	539(13),538(42),521(66),516(38),495(32),494(30),493(22),377(33),341(11),297(5),265(43),199(86),177(52)
**33**	**PA13**	13.16	C_36_H_30_O_16_Na ^b^	Salvianolic acid B ^a^	741.14142	741.14260	−1.60	561(100),543(51),517(19),363(11),362(17),319(2) ^△^
		13.18			741.14099		−2.18	579(2),561(100),543(56),517(19),363(12),362(17),361(5)
**34**	**F11**	14.21	C_28_H_33_O_14_	Buddleoside ^a^	593.18536	593.18648	−1.89	575(0.2),447(100),431(1),413(0.3),285(12),257,242 ^△^
		14.42			593.18439		−3.52	575(0.2),447(100),431(1),395(0.3),285(10),270,242
**35**	**F12**	14.23	C_16_H_13_O_5_	6,7-Dihydroxy-4’-methoxyisoflavone	285.07495	285.07575	−2.80	285(35),271(8),270(100),242(14),239(3),158(2),152(6),132(3)
**36**	**PA14**	14.42	C_18_H_15_O_8_	Prolithospermic acid	359.07535	359.07614	−2.21	341(100),315(1),313(1.4),249(3),187(1),181(21),179(25),163(11)
**37**	**PA15**	14.43	C_9_H_9_O_4_	Caffeic acid	181.04904	181.04953	−2.73	163(100),153(0.4),139(1),135(0.6),117(0.1)
**38**	**F13**	14.45	C_16_H_13_O_6_	Hispidulin	301.06982	301.07066	−2.80	301(19),286(100),269(1),241(1),183(1)
**39**	**PA16**	14.63	C_18_H_13_O_7_	Salvianolic acid G	341.06479	341.06557	−2.31	323(100),305(3),297(21),295(8),267(1),279(3),231(5),195(11),163(11)
**40**	**F14**	14.98	C_28_H_35_O_14_	Didymin	595.20074	595.20213	−2.33	577(16),559(25),541(12),449(39),433(100), 287(34)
**41**	**F15**	15.11	C_17_H_15_O_7_	Jaceosidin	331.08057	331.08122	−1.99	316(100),303(1),288(0.3),285(1),183(0.2)
**42**	**F16**	16.64	C_17_H_15_O_7_	5,6,4’-Trihydroxyl-7,8-dimethoxy flavone	331.08026	331.08122	−2.92	316(75),301(34),298(100),213(2),121(0.4)
**43**	**F17**	17.92	C_18_H_17_O_8_	Sideritiflavone	361.09082	361.09179	−2.69	347(8),346(84),331(39),328(100),300(1),213(3)
**44**	**PA17**	18.14	C_27_H_23_O_12_	*trans*-salvianolic acid J	539.11700	539.11840	−2.60	521(100),493(5),479(4),411(5),360(5),341(22),181(9),163(5)
**45**	**PA18**	18.14	C_36_H_30_O_16_Na	Sodium lithospermate B	741.14111	741.14260	−2.01	561(100),543(52),515(2),383(19),363(3),319(2)
**46**	**F18**	18.35	C_16_H_13_O_6_	Diosmetin	301.07025	301.07066	−1.37	287(3),286(100),258(1)
**47**	**T9**	18.69	C_10_H_15_O_2_	(*S*)-(−)-Perillic acid	167.10649	167.10665	−0.99	149(67),139(72),125(16),121(100),95(18),93(13)
**48**	**F19**	18.98	C_16_H_15_O_6_	Hesperetin	303.08572	303.08630	−1.96	285(13),179(35),177(100),153(20),151(3),137(1),117(1)
**49**	**PA19**	19.22	C_36_H_30_O_16_Na	Sodium lithospermate B	741.14081	741.14260	−2.42	579(37),561(14),533(100),517(7),399(3),353(4)
**50**	**PA20**	19.23	C_36_H_31_O_16_	Salvianolic acid E	719.15924	719.16066	−1.97	701(47),700(56),673(34),655(30),621(14),609(74),539(100),493(26),297(28)
**51**	**F20**	20.44	C_18_H_17_O_7_	Xanthomicrol	345.09613	345.09687	−2.17	345(15),330(100),329(85),315(1),301(14),300(3)
**52**	**F21**	21.51	C_18_H_17_O_8_	Thymonin	361.09085	361.09179	−2.61	346(100),331(75),328(53),313(33),300(13),299(4),227(1)
**53**	**F22**	22.49	C_19_H_19_O_8_	5,6-Dihydroxy-7,8,3’,4’-tetramethoxyflavone	375.10632	375.10744	−2.99	360(70),359(8),345(47),343(12),342(100),314(1),270(2),213(3),165(1)
**54**	**T10**	22.78	C_10_H_15_O_2_	(4S*)-4-hydroxy-*ρ*-mentha-1,8-dien-3-one	167.10641	167.10665	−1.47	149(100),139(100),131(13),126(35),125(47),121(84),95(26),93(14)
**55**	**T11**	24.27	C_42_H_73_O_15_	Floralquinquenoside C	817.49176	817.49439	−3.22	817(53),816(93),801(16),799(33),771(100),728(28),656(28),582(20),563(28),256(15)
**56**	**F23**	24.43	C_18_H_17_O_7_	Nevadensin	345.09686	345.09687	−0.05	330(100),315(69),312(50),301(1),297(28),284(12)
**57**	**F24**	24.72	C_19_H_19_O_8_	5,7-Dihydroxy-6,8,3’,4’-tetramethoxyflavone	375.10712	375.10744	−0.86	360(100),345(78),342(46),331(2),213
**58**	**F25**	24.78	C_16_H_13_O_5_	Acacetin	285.07047	285.07575	−0.98	285(35),271(9),270(100),243(3),242(15),152(5)
**59**	**F26**	24.99	C_18_H_17_O_7_	5,6-Dihydroxy-7,3’,4’-trimethoxy flavone	345.09662	345.09687	−0.75	330(64),315(40),312(100),284(1),240(1),213(3)
**60**	**F27**	25.08	C_16_H_13_O_5_	Genkwanin	285.07538	285.07575	−1.29	285(100),270(97),243(6),242(34),167(24),145(4)
**61**	**F28**	25.58	C_19_H_19_O_7_	5-Dydroxy-6,7,3’,4’-tetramethoxy flavones	359.11194	359.11252	−1.64	345(9),344(82),327(11),326(100),315(2),298(5),165(0.15)
**62**	**F29**	26.43	C_20_H_21_O_8_	5-Hydroxy-6,7,8,3’,4’-pentamethoxyflavone	389.12198	389.12309	−2.86	374(100),360(14),359(99),356(45),341(42),328(16),327(4),227(1.39),165(0.34)
**63**	**F30**	27.21	C_19_H_19_O_7_	Gardenin B	359.11182	359.11252	−1.97	344(100),329(92),326(53),311(37),298(15),297(5),227(1),135(1)
**64**	**T12**	28.55	C_30_H_49_O_3_	Ursolic acid	457.36615	457.36762	−3.21	439(67),411(100),393(4),356(3),227(3),191(6)

Flavonoids (F); Phenolic acids (PA); Terpenoids (T); Phenylpropanoids (P); ^a^ Identified by comparison with standards; ^△^ ESI-MS^2^ spectra of standards; ^b^ [M + Na]^+^ ions.
